# Lurasidone for the Treatment of Irritability Associated with Autistic Disorder

**DOI:** 10.1007/s10803-015-2628-x

**Published:** 2015-12-11

**Authors:** Antony Loebel, Matthew Brams, Robert S. Goldman, Robert Silva, David Hernandez, Ling Deng, Raymond Mankoski, Robert L. Findling

**Affiliations:** Sunovion Pharmaceuticals, Inc., Fort Lee, NJ USA; Sunovion Pharmaceuticals, Inc., 84 Waterford Drive, Marlborough, MA 01752 USA; Menninger Department of Psychiatry, Baylor College of Medicine, Houston, TX USA; Child and Adolescent Psychiatry, Kennedy Krieger Institute and Johns Hopkins University, Baltimore, MD USA

**Keywords:** Autism, Irritability, Lurasidone, Atypical antipsychotic

## Abstract

The aim of this study was to evaluate the short-term efficacy and safety of lurasidone in treating irritability associated with autistic disorder. In this multicenter trial, outpatients age 6–17 years who met DSM-IV-TR criteria for autistic disorder, and who demonstrated irritability, agitation, and/or self-injurious behaviors were randomized to 6 weeks of double-blind treatment with lurasidone 20 mg/day (N = 50), 60 mg/day (N = 49), or placebo (N = 51). Efficacy measures included the Aberrant Behavior Checklist Irritability subscale (ABC-I, the primary endpoint) and the Clinical Global Impressions, Improvement (CGI-I) scale, and were analyzed using a likelihood-based mixed model for repeated measures. Least squares (LS) mean (standard error [SE]) improvement from baseline to Week 6 in the ABC-I was not significantly different for lurasidone 20 mg/day (−8.8 [1.5]) and lurasidone 60 mg/day (−9.4 [1.4]) versus placebo (−7.5 [1.5]; *p* = 0.55 and 0.36, respectively). CGI-I scores showed significantly greater LS mean [SE] improvement at Week 6 for lurasidone 20 mg/day versus placebo (2.8 [0.2] vs. 3.4 [0.2]; *p* = 0.035) but not for lurasidone 60 mg/day (3.1 [0.2]; *p* = 0.27). Discontinuation rates due to adverse events were: lurasidone 20 mg/day, 4.1 %; 60 mg/day, 3.9 %; and placebo, 8.2 %. Adverse events with an incidence ≥10 % (lurasidone combined, placebo) included vomiting (18.0, 4.1 %) and somnolence (12.0, 4.1 %). Modest changes were observed in weight and selected metabolic parameters. In this study, once-daily, fixed doses of 20 and 60 mg/day of lurasidone were not demonstrated to be efficacious compared to placebo for the short-term treatment of children and adolescents with moderate-to-severe irritability associated with autistic disorder.

## Introduction

Autism spectrum disorder (ASD) is a neurodevelopmental disorder characterized by deficits in social communication and social interaction and the presence of restricted, repetitive patterns of behavior, interests, or activities. Symptoms emerge during early development and can occur with or without intellectual and/or language impairment (Lai et al. 2014; APA 2013).

ASD can be associated with a wide range of concomitant challenging behaviors (Simonoff et al. 2008). In particular, moderate to severe symptoms of irritability (broadly defined to include tantrums, aggression, self-injurious behavior, and quickly changing moods) have been observed in about a quarter of subjects in various studies (Hill et al. 2014; Lecavalier 2006). These maladaptive behaviors can interfere with everyday activities, cause substantial caregiver stress, and may have a negative impact on long-term prognosis (Bradley et al. 2004; Eisenhower et al. 2005; Murphy et al. 2005; Lecavalier et al. 2006; Volkmar et al. 1999). In addition, aggressive or self-injurious behavior is associated with an increased risk of psychiatric hospitalization among children with ASD (Mandell 2008; Siegel et al. 2012).

The atypical antipsychotics risperidone and aripiprazole are currently the only medications approved by the United States Food and Drug Administration (FDA) for the treatment of irritability associated with ASD (Volkmar et al. 2014; Carroll et al. 2014; Politte et al. 2014). Thus, there is a need to identify additional efficacious agents, especially considering the safety and tolerability issues that may be associated with use of selected antipsychotics in children (Correll et al. 2009).

Lurasidone targets both the dopamine D_2_ and serotonin 5-HT_2A_ receptor systems with a pattern of high affinity binding that is comparable to what has been reported for risperidone and aripiprazole. The receptor binding profile of lurasidone demonstrates high affinity for D_2_ (Ki, 1.68 nM; antagonist), 5-HT_1A_ (Ki, 6.74 nM; partial agonist), 5-HT_2A_ (Ki, 2.03 nM; antagonist), and 5-HT_7_ receptors (Ki, 0.495 nM; antagonist); moderate affinity for noradrenergic α_2C_ (Ki, 10.8 nM) and α_2A_ (Ki, 40.7 nM) receptors; and weak affinity for 5-HT_2C_ receptors (Ki, 415 nM; Ishibashi et al. 2010). The receptor binding profile of lurasidone has more potent affinity for the 5-HT_1A_ receptor when compared with risperidone (Ishibashi et al. 2010). In addition, lurasidone, as well as risperidone, are full antagonists at the D_2_ receptor, while aripiprazole is a partial D_2_ agonist.

Lurasidone has been approved by the US FDA for the treatment of adults with schizophrenia (Nakamura et al. 2009; Meltzer et al. 2011; Nasrallah et al. 2013), and for the treatment of bipolar I depression in adults (Loebel et al. 2014a, b).

Here, we report the results of a randomized, double-blind, placebo-controlled study to evaluate the efficacy, safety, and tolerability of lurasidone (20 or 60 mg/day) compared with placebo in treating irritability in children and adolescents with autistic disorder.

## Methods

This randomized, double-blind, fixed-dose, placebo-controlled study (ClinicalTrials.gov identifier: NCT01911442) was conducted at 40 sites in the US between September 2013 and November 2014. The study was approved by an Institutional Review Board at each investigational site and was conducted in accordance with the United States Code of Federal Regulations, the ethical principles that have their origin in the Declaration of Helsinki, and the International Conference on Harmonisation Good Clinical Practices guidelines. All parents and/or guardians provided written informed consent to participate; study subjects provided written informed assent when possible.

### Study Subjects

The study enrolled outpatients, age 6–17 years, who met DSM-IV-TR criteria for a primary diagnosis of autistic disorder (APA 2000). The diagnosis was confirmed by the Autism Diagnostic Interview, Revised (ADI-R; Lord et al. 1994) administered at the Screening Visit by an experienced clinician who had previously completed a 2-day training course conducted by an ADI-R certified trainer. Enrollment required a score ≥18 on the Irritability subscale of the Aberrant Behavior Checklist (ABC; Aman et al. 1985; Kaat et al. 2014), and a score ≥4 (moderate-or-greater severity) on the Clinical Global Impression, Severity (CGI-S; Guy 1976).

Study subjects were excluded if they had a current diagnosis of bipolar disorder, schizophrenia, major depressive disorder, Fragile-X syndrome, or childhood disintegrative disorder as confirmed by the Mini International Neuropsychiatric Interview for children and adolescents (MINI-Kid; Sheehan et al. 2010) at Screening; or a confirmed genetic disorder associated with cognitive and/or behavioral disturbance or profound intellectual disability. Study subjects were also excluded if they had a history of seizures, unless they were seizure-free and off antiepileptic drugs for at least 6 months. Concurrent behavioral therapy for autism related symptoms or behaviors was permitted if it was stable for at least 4 weeks prior to Screening, and was consistent throughout the study.

### Study Design

Study subjects who met study entry criteria were randomized, double-blind, in a 1:1:1 ratio (via an interactive voice/web response system) to receive fixed, once-daily doses of lurasidone (20 or 60 mg/day), or matching placebo, administered in the evening with a meal, or within 30 min of eating. Study subjects randomized to the 60 mg/day arm received lurasidone 20 mg/day from Days 1–3, 40 mg/day from Days 4–6, and 60 mg/day from Day 7 to Week 6. If the subject was not able to tolerate the 60 mg/day dose, a one-time dose reduction to 40 mg/day was permitted (between Day 8 and 29); the 40 mg/day dose was then maintained for the remainder of the study.

Concomitant use of psychotropic medication was prohibited, with the exception of as-needed diphenhydramine (≤50 mg/day) or melatonin (≤5 mg/day) for insomnia, benztropine (≤6 mg/day) for movement disorders, diphenhydramine (≤50 mg/day) for acute extrapyramidal symptoms (EPS), or propranolol (≤120 mg/day) for akathisia.

### Assessments

Efficacy assessments were obtained at baseline and weekly intervals. The primary efficacy measure was the caregiver-rated Aberrant Behavior Checklist Irritability subscale score (ABC-I; Aman et al. 1985; Kaat et al. 2014). The ABC is a 58-item checklist that evaluates common problem behaviors in people with developmental disorders on a 4-point severity scale. Previous factor analyses (Aman et al. 1987; Newton and Sturmey 1988; Aman et al. 1995; Ono 1996; Brown et al. 2002) have validated its five subscales: (1) irritability and agitation, (2) social withdrawal and lethargy, (3) stereotypic behavior, (4) hyperactivity and non-compliance, and (5) inappropriate speech. The ABC-I subscale consist of 15 items and ranges from 0 (no problem behaviors) to a maximum of 45. Secondary efficacy measures consisted of the other 4 subscales of the ABC, the clinician-rated Clinical Global Impression, Severity (CGI-S) and Improvement (CGI-I) scales (Guy 1976), with instructions to assess the severity and degree of improvement in irritability associated with autism; and the Children’s Yale-Brown Obsessive Compulsive Scales (CY-BOCS) modified for pervasive developmental disorders (Scahill et al. 2006). The modified CY-BOCS is a clinician-rated, semistructured assessment that eliminates the obsessions checklist and severity scales of the CY-BOCS, while expanding the compulsions checklist to include repetitive behaviors more commonly seen in children with various developmental disorders. Caregivers of the study subject were administered the Caregiver Strain Questionnaire (CGSQ; Brannan et al. 1997), which measures the degree to which the child’s condition is associated with disruption in family and community life, negative externalized emotions toward the child (anger, embarrassment), and negative internalized emotions (worry, guilt). A CGSQ global strain score is calculated by summing the three subscales and ranges from 3 to 15.

### Safety and Tolerability Evaluations

Safety and tolerability were assessed by the incidence and severity of adverse events during the study. In addition to potentially being reported as an adverse event, movement disorders were assessed in all study subjects by the Simpson-Angus Scale (SAS), the Abnormal Involuntary Movement Scale (AIMS) and the Barnes Akathisia Rating Scale (BARS; Guy 1976; Simpson and Angus 1970; Barnes 1989). Clinical chemistries (including selected metabolic parameters: glucose, cholesterol, HDL, LDL, triglycerides, hemoglobin A1c, insulin); hormonal measures (prolactin, thyrotropin and free thyroxine; testosterone [male] and serum human chorionic gonadotropin, follicle stimulating hormone, luteinizing hormone, and estradiol [female]; hematologies, urinalysis, and urine drug screen.

### Statistical Analysis

The intent-to-treat population consisted of randomized study subjects who received at least one dose of study medication and had at least one post-baseline efficacy assessment. The primary (ABC Irritability subscale) and secondary efficacy endpoints were assessed using a mixed model for repeated measures (MMRM) analysis including treatment, visit, pooled center, baseline score, and a treatment-by-visit interaction term, using an unstructured covariance for within-patient correlation. For the CGI-I analysis, a similar MMRM model without baseline as a covariate was conducted.

Criteria for CGI-I response consisted of a score ≤2 (much or very much improved) at endpoint; criteria for ABC-I response consisted of ≥25 % improvement from Baseline to Endpoint. The categorical responder variable, the ABC Irritability subscale score, was analyzed with a logistic regression model with treatment, pooled center, and corresponding baseline score as covariate. The responder outcome, based on the CGI-I score at endpoint, was analyzed using the Cochran–Mantel–Haenszel (CMH) test controlling for treatment group, and pooled center. The primary efficacy measure corrected for multiple comparisons, however, since secondary efficacy measures were not corrected, these results should be viewed as descriptive.

The safety population included all study subjects who were randomized and received at least one dose of study medication. Descriptive statistics were used to analyze safety variables including adverse events (AEs), vital signs, weight, height, body mass index (BMI), ECG, and laboratory results. In addition, a nonparametric rank ANCOVA was used to analyze selected laboratory parameters. Change from baseline to endpoint in the Simpson-Angus Scale, the Abnormal Involuntary Movement Scale and the Barnes Akathisia Rating Scale scores were analyzed using an ANCOVA model with treatment, pooled center, and baseline as covariate. To account for normal growth in a pediatric population, percentiles and z-scores for height, weight and BMI were derived (CDC 2000). A BMI z-score change <0.5 is considered not clinically significant (Correll et al. 2009).

It was estimated, based on results from two previous trials with other atypical agents (McCracken et al. 2002; Owen et al. 2009), that a sample size of 40 study participants per group would provide at least 85 % power to detect a difference from placebo as significant at the 0.05 level assuming a treatment difference of 7.0, and a common standard deviation of 11. An upward adjustment of 20 % was made to compensate for expected attrition post-randomization, yielding a total sample of 150 study participants (50 per group).

## Results

### Baseline Characteristics and Disposition

**Fig. 1:**
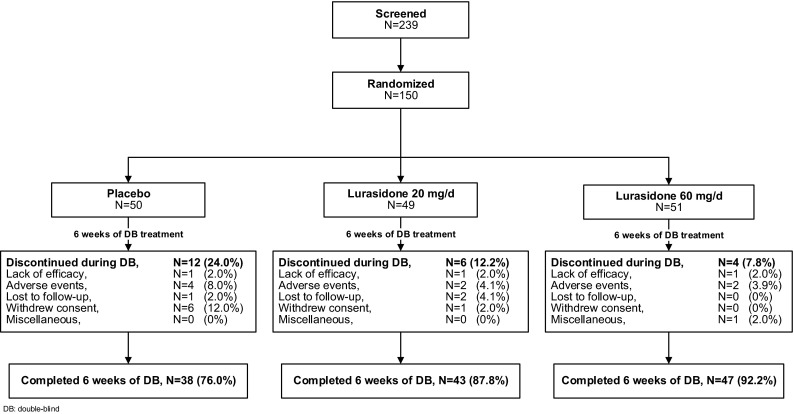
Subject disposition

A total of 150 study subjects were randomized to 6 weeks of double-blind treatment, of whom 149 received study drug (lurasidone or placebo; Fig. 1). Baseline demographic and clinical characteristics were similar across the three treatment groups (Table 1). The majority of study subjects were white (77 %), most were male (82 %); 72 % were ages 6–12 and 28 % were ages 13–17. The majority of study subjects reported previous psychotropic treatment, most commonly with an antipsychotic or a psychostimulant medication (Table 1).

**Table 1:** Baseline demographic and clinical characteristics (intent-to-treat population)

	Placebo (N = 49)	Lurasidone 20 mg/day (N = 48)	Lurasidone 60 mg/day (N = 51)
Male, n (%)	40 (81.6)	38 (79.2)	43 (84.3)
Age, years			
Mean (SD)	11 (3)	10.5 (3)	10.5 (3)
6–12, n (%)	35 (71.4)	36 (75.0)	36 (70.6)
13–17, n (%)	14 (28.6)	12 (25.0)	15 (29.4)
Race, n (%)			
White	42 (86)	34 (71)	38 (74.5)
Black	5 (10)	10 (21)	9 (17.6)
Other	2 (4)	4 (8)	4 (7.8)
Weight (kg)			
Mean (SD)	43 (14)	42 (17)	44 (17)
Percentile, mean (SD)	59 (27)	57 (30)	67 (27)
BMI, kg/m^2^			
Mean (SD)	19.2 (3.2)	18.8 (3.5)	19.2 (3.3)
Percentile, mean (SD)	61 (30)	59 (29)	64 (29)
Prior psychotropic medication, n (%)			
Any antipsychotic	19 (38.8)	17 (35.4)	16 (31.4)
Any psychostimulant	18 (36.7)	11 (22.9)	16 (31.4)
Any antidepressant	6 (12.2)	8 (16.7)	5 (9.8)
Baseline scores, mean (SD)^a^			
ABC irritability/agitation	29 (7)	28 (6)	27 (6)
CGI-severity	5.0 (0.8)	4.9 (0.8)	4.7 (0.8)

17 Subjects in the 60 mg/day dosing group received a non-protocol specified dose reduction to 40 mg/day at week 5

*ABC* aberrant behavior checklist, *BMI* body mass index, *CGI* clinical global impression, *CY-BOCS* Children’s Yale-Brown Obsessive–Compulsive Scale

^a^Intent-to-treat population

The 6-week treatment completion rates were 76 % for the placebo group, 88 % for the lurasidone 20 mg/day group, and 92 % for the lurasidone 60 mg/day group (Fig. 1).

### Efficacy

**Fig. 2:**
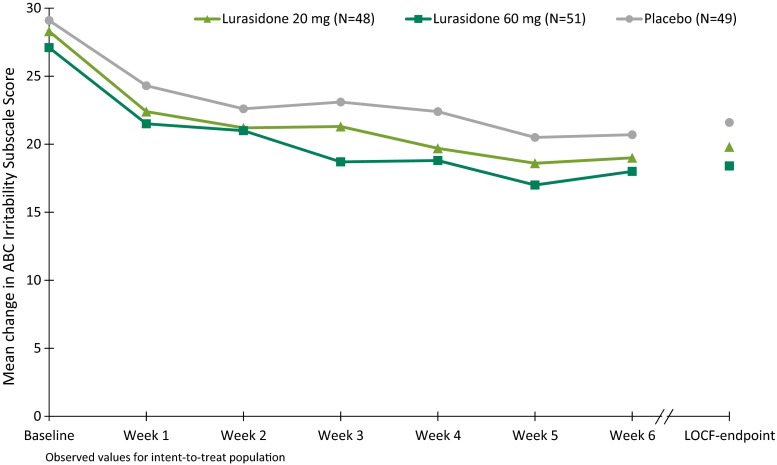
Mean change from baseline in the ABC irritability subscale score (ITT population)

The least squares (LS) mean improvement in the ABC Irritability subscale score was not significantly different for the lurasidone 20 mg/day group (−8.8) and the 60 mg/day group (−9.4) compared with placebo (−7.5) at Week 6 (Fig. 2; Table 2). Improvement in the placebo group plateaued from Weeks 2–4, and then showed additional improvement from Weeks 4 to 6; Fig. 2).

**Table 2:** Efficacy endpoints: change at week 6 (ITT population; MMRM)

	Placebo	Lurasidone 20 mg/day	Lurasidone 60 mg/day	Treatment Difference			
	(N = 49)	(N = 48)	(N = 51)	LUR-20 versus PBO (95 % CI)	*p*	LUR-60 versus PBO (95 % CI)	*p*
ABC irritability/agitation							
Baseline mean (SD)	29.1 (6.9)	28.3 (5.9)	27.1 (5.7)	−1.3 (−5.6, 3.0)	0.55	−1.9 (−6.1, 2.2)	0.36
LS mean change (SE)	−7.5 (1.5)	−8.8 (1.5)	−9.4 (1.4)				
ABC Hyperactivity, baseline							
Baseline mean (SD)	34.0 (9.2)	32.5 (8.7)	31.2 (11.3)	−2.5 (−6.8, 1.7)	0.24	+0.5 (−3.6, 4.6)	0.81
LS mean change (SE)	−7.1 (1.5)	−9.7 (1.5)	−6.6 (1.4)				
ABC stereotypic behavior							
Baseline mean (SD)	9.3 (6.3)	8.9 (5.2)	8.2 (5.1)	−1.1 (−3.0, 0.8)	0.26	+0.9 (−0.9, 2.8)	0.31
LS mean change (SE)	−2.6 (0.7)	−3.7 (0.7)	−1.6 (0.6)				
ABC inappropriate speech							
Baseline mean (SD)	7.2 (3.3)	6.8 (3.3)	6.5 (3.3)	+0.2 (−1.0, 1.4)	0.76	+0.1 (−1.1, 1.3)	0.87
LS mean change (SE)	−1.6 (0.4)	−1.4 (0.4)	−1.5 (0.4)				
ABC lethargy/withdrawal							
Baseline mean (SD)	18.7 (10.8)	15.2 (9.8)	17.4 (10.1)	−0.3 (−3.4, 2.8)	0.86	−0.9 (−3.9, 2.1)	0.55
LS mean change (SE)	−6.5 (1.1)	−6.8 (1.1)	−7.4 (1.0)				
CGI-severity							
Baseline mean (SD)	5.0 (0.8)	4.9 (0.8)	4.7 (0.8)	−0.3 (−0.8, 0.2)	0.18	−0.3 (−0.8, 0.2)	0.24
LS mean change (SE)	−0.7 (0.2)	−1.1 (0.2)	−1.0 (0.2)				
CGI-improvement^a^							
LS mean at week 6 (SE)	3.4 (0.2)	2.8 (0.2)	3.1 (0.2)	−0.6 (−1.1, −0.0)	0.035	−0.3 (−0.8, 0.2)	0.27
CY-BOCS Compulsions							
Baseline mean (SD)	12.9 (4.6)	10.7 (5.7)	10.6 (5.7)	0.2 (−1.2, 1.5)	0.82	0.2 (−1.1, 1.5)	0.73
LS mean change (SE)	−1.2 (0.5)	−1.0 (0.5)	−1.0 (0.4)				
CGSQ global strain, baseline							
Baseline mean (SD)	10.0 (1.8)	9.3 (2.5)	9.5 (2.0)	−0.1 (−1.0, 0.7)	0.75	−0.3 (−1.1, 0.5)	0.45
LS mean change (SE)	−1.4 (0.3)	−1.5 (0.3)	−1.7 (0.3)				

*MMRM* mixed model for repeated measures, *ABC* aberrant behavior checklist, *CGI* clinical global impression, *CY-BOCS* Children’s Yale-Brown Obsessive–Compulsive Scale, *CGSQ* caregiver strain questionnaire, *CI* confidence interval, *LUR* lurasidone, *PBO* placebo

^a^Total sccore (not change score)

On the CGI-Improvement score at Week 6, significant improvement was observed for the lurasidone 20 mg/day group, and numerical improvement was observed for the 60 mg/day group (Table 2). There was no significant difference at Week 6 for either dose of lurasidone compared with placebo on additional secondary efficacy measures, including other ABC subscales (hyperactivity, stereotypic behavior, inappropriate speech, lethargy/withdrawal), and on the CY-BOCS Compulsions scale, or the CGSQ Global Strain scale (Table 2). Since the secondary efficacy measures were not corrected for multiplicity, the results should be viewed as descriptive.

**Fig. 3:**
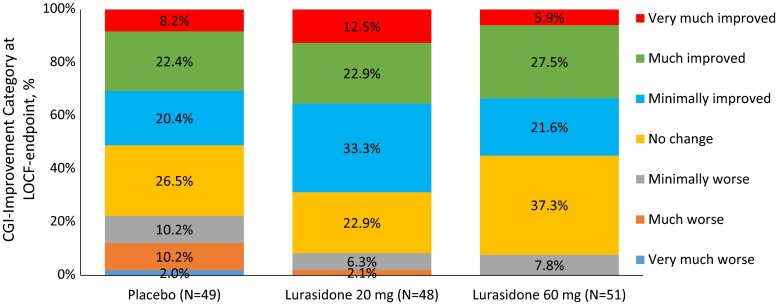
CGI-improvement category at LOCF-endpoint (ITT population)

Week 6 responder rates, using the ABC-I criterion of ≥25 % improvement from baseline, were 54.2 and 52.9 %, respectively, for lurasidone 20 and 60 mg/day, and 57.1 % for placebo (LOCF-endpoint); using a ≥50 % improvement criterion, endpoint responder rates were 31.3 and 35.3 %, respectively, for lurasidone 20 and 60 mg/day, and 22.4 % for placebo. Figure 3 summarizes the distribution of CGI-I categories at baseline and week 6.

In the 60 mg/day lurasidone arm, a non-protocol specified dose reduction, from 60 to 40 mg/day, occurred at week 5 in 17 study subjects (33 %). All 17 study subjects completed the final week of the study. Consistent with the intent to treat analysis plan, these study subjects were analyzed with the 60 mg/day dosage group to which they had been randomized. Results at week 6 on the primary efficacy outcome were similar for the dose reduction and non-dose reduction groups.

### Safety

Treatment-emergent adverse events (TEAEs) are summarized in Table 3A. The percentage of study subjects with TEAEs was 71 and 75 %, respectively, in the 20 and 60 mg/day lurasidone groups, and 57 % in the placebo group. Rates of adverse events were somewhat higher for both the 20 and 60 mg/day groups, respectively in 6–12 years old study subjects (60.0, 75.0 %), and in 13–17 years old study subjects (50.0, 67.9 %). Adverse events leading to study discontinuation were nausea and irritability (one each in the 20 mg/day group), vomiting and suicidal ideation (one each in the 60 mg/day group), and irritability, decreased appetite, disturbance in attention, psychomotor hyperactivity, and affective lability (one each in the placebo group). The majority of adverse events were rated as mild or moderate; the incidence of events rated as “severe” was 12.2 % in the lurasidone 20 mg/day group, 2.0 % in the lurasidone 60 mg/day group, and 10.2 % in the placebo group. There were 5 serious TEAEs (SAEs), 3 on the 20 mg/day dose of lurasidone (arm fractures, n = 2; increased irritability, n = 1), and 2 on the 60 mg/day dose of lurasidone (arm fracture, n = 1; appendicitis, n = 1).

**Table 3:** Tolerability and safety of lurasidone treatment (safety population)

	Placebo (N = 49)	Lurasidone 20 mg/day (N = 49)	Lurasidone 60 mg/day (N = 51)
A. *Treatment-emergent adverse events (incidence ≥5* *%* ^*a*^ *), n (%)*			
Any adverse event	28 (57)	35 (71)	38 (75)
Vomiting	2 (4)	4 (8)	14 (28)
Somnolence	2 (4)	3 (6)	9 (18)
Nasopharyngitis	0 (0)	5 (10)	3 (6)
Akathisia	0 (0)	3 (6)	3 (6)
Fatigue	1 (2)	1 (2)	4 (8)
Weight increased	1 (2)	1 (2)	4 (8)
Cough	2 (4)	2 (4)	3 (6)
Sedation	1 (2)	3 (6)	1 (2)
Constipation	1 (2)	0 (0)	3 (6)
Nausea	0 (0)	2 (4)	3 (6)
B. *Change in weight, BMI, and fasting laboratory parameters (week 6* ^*b*^ *)*			
Weight, kg			
LS mean (SE) change	+0.4 (0.2)	+0.5 (0.2)	+1.2 (0.2)^d^
Mean (SD) change in percentile	−0.9 (6.6)	+0.8 (5.4)	+2.7 (6.5)
LS mean (SE) z-score change	−0.02 (0.03)	+0.02 (0.03)	+0.09 (0.03)
BMI (kg/m^2^)			
LS mean (SE) change	−0.0 (0.1)	−0.04 (0.1)	+0.4 (0.1)
Mean (SD) change in percentile	−1.1 (7.6)	+0.3 (6.5)	+3.3 (9.5)
LS mean (SE) z-score change	−0.02 (0.04)	−0.02 (0.04)	+0.1 (0.04)
Waist circumference, cm, mean (SD) change	+0.5 (3.0)	+0.2 (1.6)	+1.1 (2.6)
Cholesterol (mg/dL, median change)^c^	−5.0	+6.0	+7.5
Triglycerides (mg/dL, median change)^c^	−4.0	+1.0	+15.0
Glucose (mg/dL, median change)^c^	−5.0	−1.0	−1.0
HbA1c (%, mean (SD) change)	+0.0 (0.3)	+0.1 (0.2)	+0.1 (0.4)
Prolactin (ng/mL, mean (SD) change)	−0.1 (5.9)	−0.2 (9.0)	+2.3 (13.9)

*BMI* body mass index, *HbA1c* glycosylated hemoglobin

^a^Adverse events shown where incidence on lurasidone > placebo

^b^Endpoint data, except for weight and BMI, which were analyzed by MMRM

^c^Fasting subjects: placebo (n = 36); lurasidone 20 mg/day (n = 37); lurasidone 60 mg/day (n = 45)

^d^ *p* value (vs. placebo): 0.015

Treatment with lurasidone (20 and 60 mg/day vs. placebo) was associated with small mean changes at the Week 6 endpoint in the BARS total score (+0.08 and −0.12 vs. +0.00) and the SAS 10-item mean score (−0.01 and −0.05 vs. −0.01); no shift from normal to abnormal in the AIMS total score were observed in either of the two lurasidone groups, while one study subject shifted from normal to abnormal in the placebo group. The only EPS symptom reported by more than one study subject in a treatment group was akathisia (Table 3A). No concomitant anti-Parkinsonian medication or benzodiazepines were used by study subjects in either of the three treatment groups.

Increased weight was observed in all three treatment groups at Week 6 (Table 3B). The increased weight was similar for lurasidone 20 mg/day compared with placebo, but a greater increase was noted for the lurasidone 60 mg/day group. The mean z-score change in both weight and BMI were similar for lurasidone 20 mg/day and 60 mg/day versus placebo (−0.02 and +0.1 vs. −0.02). Six weeks of treatment with lurasidone was associated with minimal changes in laboratory parameters compared with placebo, with the exception of an increase for the lurasidone 60 mg/day group versus placebo in triglycerides (median change, +13.0 vs. −4.0 mg/dL) and cholesterol (median change, +8.0 vs. −5.0 mg/dL). No clinically meaningful effect on vital signs or ECG parameters were observed; changes at Week 6 in QTcF were +0.3 ms, −1.1 ms, and +3.2 ms, respectively, for the placebo, lurasidone 20, and 60 mg/day groups. No study subjects treated with lurasidone had clinically significant ECG abnormalities.

## Discussion

In this randomized, double-blind, fixed-dose (20, 60 mg/day), 6-week study, lurasidone did not significantly differentiate from placebo on the primary endpoint, change in the ABC Irritability subscale. Significantly greater improvement was observed at endpoint on the CGI-I scale for the lurasidone 20 mg/day group compared with the placebo group. However, no significant difference was observed for either dose of lurasidone compared with placebo on other secondary efficacy measures at either dose.

The precise pathophysiology of irritable, aggressive and/or self-injurious behavior in autistic disorder has not been determined. Abnormal serotonergic and/or dopaminergic neurotransmission has been hypothesized to be related to this constellation of behaviors, which suggests a potential therapeutic role for atypical antipsychotics (Lesch and Merschdorf 2000; Moore et al. 2002; Siever 2008; Seo et al. 2008; Callesen et al. 2013; Duke et al. 2013; Kolevzon et al. 2014).

Risperidone and aripiprazole have demonstrated efficacy in the treatment of irritability associated with autistic disorder (McCracken et al. 2002; Owen et al. 2009; Marcus et al. 2009). Risperidone, aripiprazole, and lurasidone target both the dopamine D_2_ and serotonin (5-HT) receptor systems with a comparable pattern of high affinity binding (Ishibashi et al. 2010; Gründer et al. 2006). Furthermore, experience in clinical practice suggests that lurasidone may be useful for the treatment of irritability associated with autistic disorder (Millard et al. 2014). However, the results of the current study did not confirm the efficacy of lurasidone in this population.

The reasons for the negative results of the current study are uncertain. Differences in study populations between this study and the aripiprazole and risperidone studies do not appear to account for the negative findings. Baseline characteristics of the current study population were similar to previously reported positive short-term trials of risperidone (McCracken et al. 2002; McDougle et al. 2005) and aripiprazole (Owen et al. 2009; Marcus et al. 2009), with one notable exception: a somewhat higher proportion of study subjects in the current trial reported a history of prior treatment with antipsychotics (35 %) compared with clinical trials of risperidone (6 %) and aripiprazole (21 %). It is also possible that subtle differences in the pharmacology of lurasidone compared with risperidone and aripiprazole may account for the lack of observed efficacy in the current trial.

Based on the level of improvement observed on placebo, the current study population did not appear to be notably treatment-resistant. Week 6 improvement in the ABC-I on placebo in the current study (−7.5) was larger than has been reported for risperidone (−3.5; McCracken et al. 2002) and for one of the aripiprazole trials (−5.0; Marcus et al. 2009) but not for the other aripiprazole trial (−8.4; Owen et al. 2009). Finally, it is possible that the lack of flexible dosing might have reduced the ability to detect an efficacy signal.

Discontinuations due to adverse events were lower in both lurasidone groups compared with placebo. Treatment-emergent adverse events were typically mild-to-moderate in severity. Only vomiting and somnolence showed apparent substantial dose-related increases in event rates. The 20 mg/day dose of lurasidone was generally similar to placebo in its effects on weight, metabolic parameters, and prolactin. The 60 mg/day dose of lurasidone was associated with increased effects on weight and lipids (but not glycemic indices), and prolactin. As with all study participants, especially younger ones, clinicians should be mindful of potential weight and metabolic changes that can occur during treatment with an atypical antipsychotic, though different antipsychotics have demonstrated different metabolic risk profiles (Correll et al. 2015; Galling and Correll 2015).

Several potential study limitations should be noted. First is the absence of an active (risperidone or aripiprazole) control group. Inclusion of an active control group is the only reliable method for determining whether a treatment-responsive sample has been recruited, or whether the assay was defective, and the study was a failed trial rather than a negative trial. Second, the study design did not include a single-blind, placebo run-in period, which may have served to reduce the placebo response rate. Third, no formal cognitive assessment of intellectual functioning was obtained. Patients with profound intellectual disability, based on investigator judgment, were excluded from study entry, however, the contribution of baseline intellectual functioning to study outcome could not be ascertained. Finally it should be noted that relatively few placebo-controlled clinical trials have been conducted in irritability associated with autistic disorder. Therefore, our confidence in the reliability and validity of the outcome measures, and the sample size required to detect a treatment effect are not nearly as well established as they are for other disorders.

In conclusion, in this randomized, placebo-controlled 6-week study, lurasidone did not demonstrate statistically significant efficacy for the treatment of irritability associated with autistic disorder. The safety profile of lurasidone was consistent with the safety profile in adults, with the exception of some weight gain seen at the higher dose in this pediatric population.

## Clinical Significance

In this randomized, placebo-controlled 6-week study, treatment with a fixed dose of lurasidone (20 or 60 mg/day) was not found to be significantly superior to placebo in reducing moderate-to-severe irritability in children and adolescents with a diagnosis of autistic disorder. Although some individual study subjects had meaningful improvements in symptoms, the lack of statistical significance on the primary outcome measure compared with placebo is in contrast to significant efficacy previously reported for two other atypical antipsychotics, risperidone and aripiprazole, both of which are FDA approved for this use.
